# Author Correction: CFTR function, pathology and pharmacology at single-molecule resolution

**DOI:** 10.1038/s41586-023-06115-3

**Published:** 2023-05-04

**Authors:** Jesper Levring, Daniel S. Terry, Zeliha Kilic, Gabriel Fitzgerald, Scott C. Blanchard, Jue Chen

**Affiliations:** 1 Laboratory of Membrane Biology and Biophysics, The Rockefeller University, New York, NY USA; 2 Department of Structural Biology, St. Jude Children’s Research Hospital, Memphis, TN USA; 3 Department of Physiology and Biophysics, Weill Cornell Medicine, New York, NY USA; 4 Howard Hughes Medical Institute, The Rockefeller University, New York, NY USA

**Keywords:** Chloride channels, Membrane proteins, Single-molecule biophysics, Cryoelectron microscopy, Kinetics

Correction to: *Nature* 10.1038/s41586-023-05854-7 Published online 16 February 2023

In the version of this article initially published, the name of Scott C. Blanchard was missing the middle initial. The omission has been corrected in the HTML and PDF versions of the article.

