# Supplementary material for: CFTR function, pathology and pharmacology at single-molecule resolution
Source: Nature. 2023 Mar 22;616(7957):606–14. doi: 10.1038/s41586-023-05854-7 (PMC10115640; doi:10.1038/s41586-023-05854-7)
Supplement: Supplementary file 1 — This file contains Supplementary Methods, Tables 1 and 2, Fig. 1 and References. [file 41586_2023_5854_MOESM1_ESM.pdf]

nature portfolio

https://doi.org/10.1038/s41586-023-05854-7

Supplementary information

CFTR function, pathology and pharmacology at single-molecule resolution

In the format provided by the authors and unedited

CFTR function, pathology and pharmacology at single-molecule resolution

Jesper Levring[1], Daniel S. Terry[2], Zeliha Kilic[2], Gabriel Fitzgerald[3], Scott Blanchard[2,*] & Jue Chen[1,4,*]

[1]Laboratory of Membrane Biology and Biophysics, The Rockefeller University, 1230 York Ave, New York, NY, USA.

[2]Department of Structural Biology, St. Jude Children's Research Hospital, Memphis, TN, USA.

[3]Department of Physiology and Biophysics, Weill Cornell Medicine, 1300 York Ave, New York, NY, USA.

[4]Howard Hughes Medical Institute, The Rockefeller University, 1230 York Ave, New York, NY, USA.

*To whom correspondence should be addressed: Scott.Blanchard@stjude.org, juechen@rockefeller.edu.

Supplementary Methods

Calculations of the steady state population fractions

Here, we demonstrate the kinetic scheme formulation at physiologically relevant saturating ATP concentrations ($>$ 1 mM) denoted with [ATP] starting with the state occupation probabilities when the system is at steady state. The population fractions of the states are labeled with S_l for $l = 1,2,\ldots,8$. Temporal evolution of these population fractions is governed by the following set of equations where the transition rates from state l to l' are shown with $k_{l\rightarrow l'}$ for all $l \neq l' = 1,2,\ldots,8$

$$\frac{dS_1}{dt} = -\left(k_{1\rightarrow 2}\,[ATP] + k_{1\rightarrow 8}\right)S_1 + k_{2\rightarrow 1}S_2 + k_{8\rightarrow 1}S_8,$$

$$\frac{dS_2}{dt} = -\left(k_{2\rightarrow 1} + k_{2\rightarrow 3}\right)S_2 + k_{3\rightarrow 2}S_3 + k_{1\rightarrow 2}[ATP]S_1,$$

$$\frac{dS_3}{dt} = -\left(k_{3\rightarrow 8} + k_{3\rightarrow 2} + k_{3\rightarrow 4}\right)S_3 + k_{2\rightarrow 3}S_2 + k_{4\rightarrow 3}S_4 + k_{8\rightarrow 3}[ATP]S_8,$$

$$\frac{dS_4}{dt} = -\left(k_{4\rightarrow 3} + k_{4\rightarrow 5}\right)S_4 + k_{3\rightarrow 4}S_3 + k_{5\rightarrow 4}S_5,$$

$$\frac{dS_5}{dt} = -\left(k_{5\rightarrow 4} + k_{5\rightarrow 6}\right)S_5 + k_{4\rightarrow 5}S_4 + k_{6\rightarrow 5}S_6,$$

$$\frac{dS_6}{dt} = -\left(k_{6\rightarrow 5} + k_{6\rightarrow 7}\right)S_6 + k_{7\rightarrow 6}S_7 + k_{5\rightarrow 6}S_5,$$

$$\frac{dS_7}{dt} = -\left(k_{7\rightarrow 6} + k_{7\rightarrow 8}\right)S_7 + k_{6\rightarrow 7}S_6 + k_{8\rightarrow 7}S_8,$$

$$\frac{dS_8}{dt} = -\left(k_{8\rightarrow 3}\,[ATP] + k_{8\rightarrow 7} + k_{8\rightarrow 1}\right)S_8 + k_{1\rightarrow 8}S_1 + k_{8\rightarrow 1}S_8,$$

$$\sum_{k=1}^{8} S_k = 1.$$

Here, the last equation imposes conservation of mass on the population fractions. We find the steady state solution for the state population fractions. steady state population fractions is used to initialize our data simulation. Below we provide the details of the data simulation.

Simulation of simultaneous electrophysiology and smFRET data

In our simulation setup, we imagine visualizing the single molecule conformations with smFRET and electrophysiology, simultaneously. As the single molecule transitions amongst its conformational states labeled with S_l for $l = 1,2,\dots,8$ where some of these states give rise the same signal in smFRET or electrophysiology measurements as summarized below in **Supplementary Table 1** with kinetic rates provided in **Supplementary Table 2.**

Supplementary Table 1. Conformational states and their associated signals in smFRET and electrophysiology measurements.

occupied conformational state	smFRET signal	electrophysiology signal
S_1	low FRET	no current
S_2	low FRET	no current
S_3	high FRET	no current
S_4	high FRET	no current
S_5	high FRET	current
S_6	high FRET	current
S_7	high FRET	no current
S_8	high FRET	no current

Here, we simulate the trajectory reporting on the conformational dynamics of the single molecule in continuous time as a Gillespie[60] trajectory where these conformational dynamics are governed by the following generator matrix labeled with $\bar{\bar{G}}$

$$
\begin{array}{ccccccccc}
 & S_1 & S_2 & S_3 & S_4 & S_5 & S_6 & S_7 & S_8 \\
S_1 & * & k_{1\rightarrow 2}[ATP] & 0 & 0 & 0 & 0 & 0 & k_{1\rightarrow 8} \\
S_2 & k_{2\rightarrow 1} & * & k_{2\rightarrow 3} & 0 & 0 & 0 & 0 & 0 \\
S_3 & 0 & k_{3\rightarrow 2} & * & k_{3\rightarrow 4} & 0 & 0 & 0 & k_{3\rightarrow 8} \\
S_4 & 0 & 0 & k_{4\rightarrow 3} & * & k_{4\rightarrow 5} & 0 & 0 & 0 \\
S_5 & 0 & 0 & 0 & k_{5\rightarrow 4} & * & k_{5\rightarrow 6} & 0 & 0 \\
S_6 & 0 & 0 & 0 & 0 & k_{6\rightarrow 5} & * & k_{6\rightarrow 7} & 0 \\
S_7 & 0 & 0 & 0 & 0 & 0 & k_{7\rightarrow 6} & * & k_{7\rightarrow 8} \\
S_8 & k_{8\rightarrow 1} & 0 & k_{8\rightarrow 3}[ATP] & 0 & 0 & 0 & k_{8\rightarrow 7} & *
\end{array}
$$

where the $*$ notation shows the negative of the sum of each row.

Subsequently, this trajectory of the single molecule gives rise to smFRET and electrophysiology measurements. For example, if the molecule has dimerized NBDs and an open pore then this conformation of the molecule leads to high FRET and high current measurements in smFRET and electrophysiology, respectively.

In a prototypical smFRET experiment, FRET efficiency (FRET) is computed based on the photons emitted from donor (n_D) and acceptor dyes (n_A) that are attached to the single molecule where $FRET = \frac{n_A}{n_A+n_D}$.

In our simulations, we adjusted the photon emission rates of donor and acceptor dyes according to known total experimentally observed photon counts such that the low and high FRET signals are equivalent to ~20 % and ~55 % FRET, respectively. For the electrophysiology measurements, based on the occupied states of the single molecule's trajectory, we generate measurements. Specifically, low (closed channel conformation) and high (open channel conformation) current levels are adjusted according to the experimentally observed conformational state levels in electrophysiology experiments where low and high current levels are ~0 pA and ~2 pA. Both smFRET and electrophysiology measurements are acquired with a camera-based detector[61] thereby the measurements are subject to temporal averaging provided that the conformational dynamics of the molecule are faster than the data acquisition rate labeled with $\frac{1}{\Delta t}$ where Δt denotes the data acquisition period. We use Δt^F and Δt^E for smFRET and electrophysiology experiments, respectively. Here, we denote the trajectory of the molecule with $\tau(t)$, for all $t \geq 0$, where photon emission

rates for donor and acceptor dyes that lead to low and high FRET levels are denoted with $\bar{\mu}_D = (\mu_1^D, \mu_2^D)$and $\bar{\mu}_A = (\mu_1^A, \mu_2^A)$. We also note that for a given time t, $\tau(t) = S_l$ for some $l = 1,2,\ldots,8$. Low and high current state levels for the electrophysiology measurements are denoted with $\bar{C} = (C_1, C_2)$. We assumed shot noise for the smFRET measurements and normally distributed measurement noise for the electrophysiology experiments.

Now, we summarize how simulated measurements in smFRET and electrophysiology experiments are generated.

Based on Gillespie simulation, we generate $\tau(.) \sim Gillespie(\bar{\bar{G}}, \tau(0))$, where $'\sim'$ stands for sampling of $\tau(.)$ as a Gillespie trajectory with a given starting point denoted with $\tau(0)$ at time $t = 0$ that is determined based on the steady state probability of state occupation explained above. Subsequently, given the photon emission rates $\bar{\mu}_D$ and $\bar{\mu}_A$ of donor and acceptor dyeswe can now formulate the collected photons associated with donor and acceptor dyes. The measurements are labeled with $\bar{w}_D = (w_1^D, w_2^D, \ldots, w_N^D)\ and\ \bar{w}_A = (w_1^A, w_2^A, \ldots, w_N^A)$ on the donor and acceptor dyes associated detectors in smFRET experimental setup where w_n^D, w_n^A denote the n-th measurementfor all $n = 1,2,\ldots,N$ with N labeling the last smFRET measurement. Then, we now formulate the n-th measurement during the data acquisition period Δt^F within the time window of $[t_{n-1}, t_{n-1} + \Delta t^F]$ such that $w_n^D \sim Poisson(\int_{t_{n-1}}^{t_{n-1}+\Delta t^F} dt\, \mu_{\tau(t)}^D)$ and $w_n^A \sim Poisson(\int_{t_{n-1}}^{t_{n-1}+\Delta t^F} dt\, \mu_{\tau(t)}^A)$ for all $n = 1,\ldots,N$. Here, "Poisson" denotes the distribution to formulate the shot noise statistics in the measurements. Next, we move to the electrophysiology measurements labeled with $\bar{y} = (y_1, y_2, \ldots, y_M)$ where M labels the last measurement in electrophysiology experiments. In the case of acquiring electrophysiological data, we assume that the measurement noise is governed by "Normal" distribution and thereby the m-th measurement labeled with y_m collected with data acquisition rate $\frac{1}{\Delta t^E}$ within the time window $[t_{m-1}, t_{m-1} + \Delta t^E]$ shown as

$y_m \sim Normal(\frac{1}{\Delta t^E}\int_{t_{m-1}}^{t_{m-1}+\Delta t^E} dt\ C_{\tau(t)}), \sigma^2)$ for all $m = 1,2,\ldots,M$ where σ^2 (pA^2) denotes the measurement noise.

In our current study, the only noise sources are the shot noise due to photon emissions for smFRET and normally distributed measurement noise for electrophysiology. We have not considered the read-out or any jittering noises dictated by the type of detector used in the experiments. Those additional improvements related to the measurement models in our simulations that are left for future studies.

Supplementary Table 2. Kinetic rate parameters used in simulations. *For simulation of E1371Q gating $k_{5\rightarrow 6}$ was set to zero.

rates	units	values
$k_{1\rightarrow 2}$	$\mu M^{-1}s^{-1}$	1
$k_{2\rightarrow 1}$	s^{-1}	50
$k_{2\rightarrow 3}$	s^{-1}	10
$k_{3\rightarrow 2}$	s^{-1}	0.3
$k_{1\rightarrow 8}$	s^{-1}	0.3
$k_{8\rightarrow 1}$	s^{-1}	0.3
$k_{3\rightarrow 8}$	s^{-1}	0.6
$k_{8\rightarrow 3}$	$\mu M^{-1}s^{-1}$	0.012
$k_{3\rightarrow 4}$	s^{-1}	1.3
$k_{4\rightarrow 3}$	s^{-1}	0.002
$k_{5\rightarrow 4}$	s^{-1}	20
$k_{4\rightarrow 5}$	s^{-1}	100
$k_{5\rightarrow 6}$	s^{-1}	2*
$k_{6\rightarrow 5}$	s^{-1}	0
$k_{6\rightarrow 7}$	s^{-1}	4
$k_{7\rightarrow 6}$	s^{-1}	0
$k_{7\rightarrow 8}$	s^{-1}	4.5
$k_{8\rightarrow 7}$	s^{-1}	0

REFERENCES:

60. Gillespie, D. T. (1977). Exact stochastic simulation of coupled chemical reactions. *The journal of physical chemistry*, *81*(25), 2340-2361.

61. Pati, A. K., El Bakouri, O., Jockusch, S., Zhou, Z., Altman, R. B., Fitzgerald, G. A., ... & Blanchard, S. C. (2020). Tuning the Baird aromatic triplet-state energy of cyclooctatetraene to maximize the self-healing mechanism in organic fluorophores. *Proceedings of the National Academy of Sciences*, *117*(39), 24305-24315.

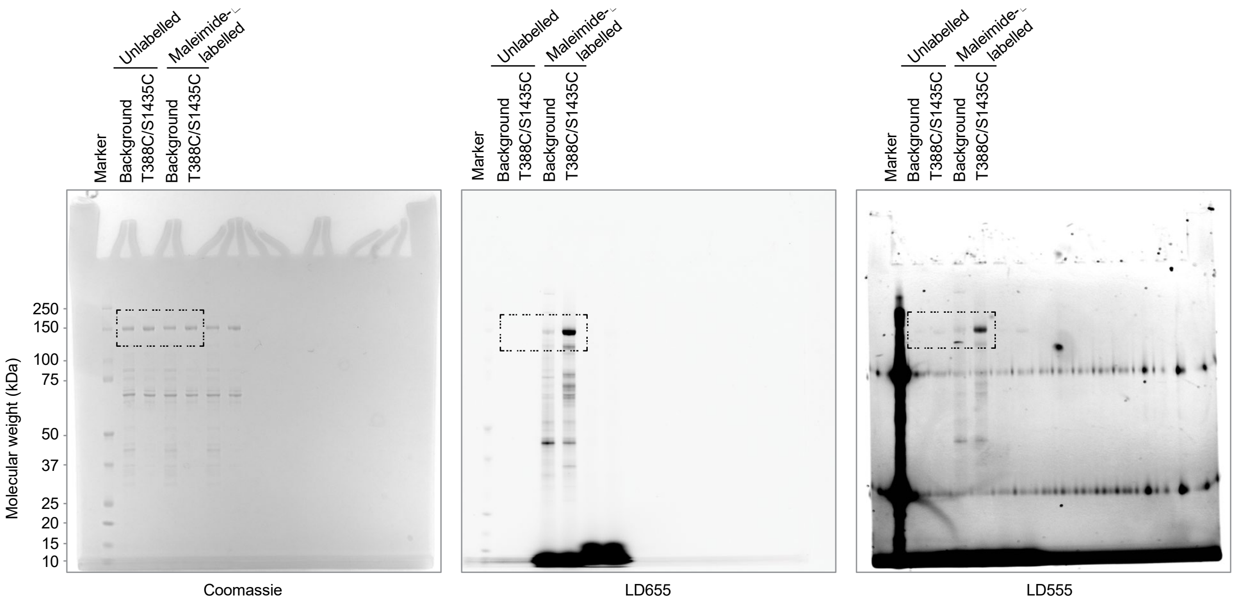

Supplementary Figure 1 | Gel source data
Uncropped gel scans of the gel imaged in Extended Data Figure 2a. The gel was first imaged for LD555 and LD655 fluorescence, and then Coomassie-stained. The dashed boxes indicate the regions of the scans that are presented in Extended Data Figure 2a.
