# Supplementary material for: CFTR function, pathology and pharmacology at single-molecule resolution
Source: Nature. 2023 Mar 22;616(7957):606–14. doi: 10.1038/s41586-023-05854-7 (PMC10115640; doi:10.1038/s41586-023-05854-7)
Supplement: Supplementary file 2 — Reporting Summary [file 41586_2023_5854_MOESM2_ESM.pdf]

nature portfolio

Corresponding author(s): Scott C. Blanchard & Jue Chen

Last updated by author(s): 2023/01/31

Reporting Summary

Nature Portfolio wishes to improve the reproducibility of the work that we publish. This form provides structure for consistency and transparency in reporting. For further information on Nature Portfolio policies, see our Editorial Policies and the Editorial Policy Checklist.

Statistics

For all statistical analyses, confirm that the following items are present in the figure legend, table legend, main text, or Methods section.

n/a	Confirmed	
☐	☒	The exact sample size (*n*) for each experimental group/condition, given as a discrete number and unit of measurement
☒	☐	A statement on whether measurements were taken from distinct samples or whether the same sample was measured repeatedly
☐	☒	The statistical test(s) used AND whether they are one- or two-sided<br>*Only common tests should be described solely by name; describe more complex techniques in the Methods section.*
☐	☒	A description of all covariates tested
☒	☐	A description of any assumptions or corrections, such as tests of normality and adjustment for multiple comparisons
☐	☒	A full description of the statistical parameters including central tendency (e.g. means) or other basic estimates (e.g. regression coefficient) AND variation (e.g. standard deviation) or associated estimates of uncertainty (e.g. confidence intervals)
☐	☒	For null hypothesis testing, the test statistic (e.g. *F*, *t*, *r*) with confidence intervals, effect sizes, degrees of freedom and *P* value noted<br>*Give P values as exact values whenever suitable.*
☒	☐	For Bayesian analysis, information on the choice of priors and Markov chain Monte Carlo settings
☒	☐	For hierarchical and complex designs, identification of the appropriate level for tests and full reporting of outcomes
☒	☐	Estimates of effect sizes (e.g. Cohen's *d*, Pearson's *r*), indicating how they were calculated

Our web collection on statistics for biologists contains articles on many of the points above.

Software and code

Policy information about availability of computer code

Data collection	Software used are commercial products: Labview 2017, Clampex 10.7, SerialEM 3.7.
Data analysis	All software used are commercial products or otherwise publicly available: Chimera 1.13.1, Clampfit 10.7, Coot 0.8.9.2, GraphPad Prism 8, ImageJ 2.0, Matlab R2019a, Motioncorr2, OriginPro 2022, Pymol2, Relion-3, and SPARTAN.

For manuscripts utilizing custom algorithms or software that are central to the research but not yet described in published literature, software must be made available to editors and reviewers. We strongly encourage code deposition in a community repository (e.g. GitHub). See the Nature Portfolio guidelines for submitting code & software for further information.

Data

Policy information about availability of data

All manuscripts must include a data availability statement. This statement should provide the following information, where applicable:

- Accession codes, unique identifiers, or web links for publicly available datasets
- A description of any restrictions on data availability
- For clinical datasets or third party data, please ensure that the statement adheres to our policy

Structural models in the RCSB PDB under accession codes 5UAK, 6MSM, and 6O2P were used in this study.
The CFTR2 database was used in this study.
The cryo-EM map has been deposited in the Electron Microscopy Data Bank (EMDB) under accession code: EMD-29637. The corresponding atomic model has been

deposited in the Protein Data Bank (PDB) under accession code 8FZQ.
The data that support the findings are available from the authors upon reasonable request.

Human research participants

Policy information about studies involving human research participants and Sex and Gender in Research.

Reporting on sex and gender	N/A
Population characteristics	N/A
Recruitment	N/A
Ethics oversight	N/A

Note that full information on the approval of the study protocol must also be provided in the manuscript.

Field-specific reporting

Please select the one below that is the best fit for your research. If you are not sure, read the appropriate sections before making your selection.

☒ Life sciences ☐ Behavioural & social sciences ☐ Ecological, evolutionary & environmental sciences

For a reference copy of the document with all sections, see nature.com/documents/nr-reporting-summary-flat.pdf

Life sciences study design

All studies must disclose on these points even when the disclosure is negative.

Sample size	Statistical methods were not used to determine sample sizes. Sample sizes were determined by the normal throughput of the instrumentation while ensuring that three or more independent replicates were performed for statistical analysis. For single molecule FRET experiments, each replicate reflects approximately 300 to 2000 analyzed trajectories.
Data exclusions	Single molecule FRET trajectories were selected for analysis based on the following pre-established criteria: (1) single-step donor photobleaching, (2) a signal-to-noise ratio > 8, (3) fewer than four donor blinking events, and (4) FRET efficiency above baseline for at least 50 frames. Details on data exclusion criteria are described in the manuscript.<br>Further, single molecule traces exhibiting FRET values above 0.8 were excluded from analysis. This sub-population was insensitive to phosphorylation and nucleotide addition, and likely reflected denatured molecules. This criterion was not pre-established.<br>Details on data exclusion can also be found in the manuscript.
Replication	Findings were reliably replicated across different preparations. The number of experimental replicates are specified in the legends of all figures.
Randomization	This study did not allocate experimental groups thus no randomization was required for the reported experiments.
Blinding	Blinding was not performed. No a priori knowledge could be assumed about the present observations and blinding is therefore not applicable. Data was analyzed systematically as described in the manuscript.

Reporting for specific materials, systems and methods

We require information from authors about some types of materials, experimental systems and methods used in many studies. Here, indicate whether each material, system or method listed is relevant to your study. If you are not sure if a list item applies to your research, read the appropriate section before selecting a response.

Materials & experimental systems

n/a	Involved in the study	
☐	☒	Antibodies
☐	☒	Eukaryotic cell lines
☒	☐	Palaeontology and archaeology
☒	☐	Animals and other organisms
☒	☐	Clinical data
☒	☐	Dual use research of concern

Methods

n/a	Involved in the study	
☒	☐	ChIP-seq
☒	☐	Flow cytometry
☒	☐	MRI-based neuroimaging

Antibodies

Antibodies used	This study used an anti-GFP nanobody that was initially described in:<br>Kirchhofer, A., Helma, J., Schmidthals, K. et al. Modulation of protein properties in living cells using nanobodies. Nat Struct Mol Biol 17, 133–138 (2010).
Validation	The nanobody was previously used for the same application as described in:<br>Liu, F., Zhang, Z., Csanády, L., Gadsby, D. C., & Chen, J. Molecular structure of the human CFTR ion channel. Cell 169, 85–95 (2017).<br>Zhang, Z., Liu, F., & Chen, J. Molecular structure of the ATP-bound, phosphorylated human CFTR. Proc Natl Acad Sci USA 115, 12757–12762 (2018).<br>Liu, F., et al. Structural identification of a hotspot on CFTR for potentiation. Science, 364, 1184–1188 (2019).

Eukaryotic cell lines

Policy information about cell lines and Sex and Gender in Research

Cell line source(s)	CHOK1 (CCL-61, lot number 70014310) and HEK293S GnTI- (CRL-3022, lot number 62430067) cells were acquired from the American Type Culture Collection (ATCC). Sf9 cells (catalog number 11496015, lot number 1670337) were acquired from GIBCO.
Authentication	CHOK1 and HEK293S GnTI- cells were authenticated by the American Type Culture Collection (ATCC). Details of authentication are outlined by the vendor.<br>CHOK1 cells: https://www.atcc.org/products/ccl-61#generalinformation. Specifically, CHOK1 cells were visually inspected for appropriate morphoplogy, validated to be the correct species by a COI assay, and tested for contamination of bacteria and mycoplasma.<br>HEK293S GnTI- cells: https://www.atcc.org/products/crl-3022. Specifically, HEK293S GnTI- cells were visually inspected for appropriate morphology, validated to be the correct species by a COI assay and STR analysis, and tested for contamination of bacteria, mycoplasma, and human pathogenic viruses.<br>Sf9 cells were authenticated by the vendor as outlined at: https://www.thermofisher.com/order/catalog/product/11496015. Sepcifically, Sf9 cells were tested for contamination of bacteria, yeast, mycoplasma and virus and were characterized by isozyme and karyotype analysis.
Mycoplasma contamination	Sf9 and HEK293S GnTI- cells tested negative for Mycoplasma contamination. CHOK1 cells were not tested for mycoplasma contamination.
Commonly misidentified lines<br>(See ICLAC register)	No commonly misidentified cell lines were used in this study.
